# sAMPpred-GAT: prediction of antimicrobial peptide by graph attention network and predicted peptide structure

**DOI:** 10.1093/bioinformatics/btac715

**Published:** 2022-11-07

**Authors:** Ke Yan, Hongwu Lv, Yichen Guo, Wei Peng, Bin Liu

**Affiliations:** School of Computer Science and Technology, Beijing Institute of Technology, Beijing 100081, China; School of Computer Science and Technology, Beijing Institute of Technology, Beijing 100081, China; School of Computer Science and Technology, Beijing Institute of Technology, Beijing 100081, China; School of Computer Science and Technology, Beijing Institute of Technology, Beijing 100081, China; School of Computer Science and Technology, Beijing Institute of Technology, Beijing 100081, China; Advanced Research Institute of Multidisciplinary Science, Beijing Institute of Technology, Beijing 100081, China

## Abstract

**Motivation:**

Antimicrobial peptides (AMPs) are essential components of therapeutic peptides for innate immunity. Researchers have developed several computational methods to predict the potential AMPs from many candidate peptides. With the development of artificial intelligent techniques, the protein structures can be accurately predicted, which are useful for protein sequence and function analysis. Unfortunately, the predicted peptide structure information has not been applied to the field of AMP prediction so as to improve the predictive performance.

**Results:**

In this study, we proposed a computational predictor called sAMPpred-GAT for AMP identification. To the best of our knowledge, sAMPpred-GAT is the first approach based on the predicted peptide structures for AMP prediction. The sAMPpred-GAT predictor constructs the graphs based on the predicted peptide structures, sequence information and evolutionary information. The Graph Attention Network (GAT) is then performed on the graphs to learn the discriminative features. Finally, the full connection networks are utilized as the output module to predict whether the peptides are AMP or not. Experimental results show that sAMPpred-GAT outperforms the other state-of-the-art methods in terms of AUC, and achieves better or highly comparable performance in terms of the other metrics on the eight independent test datasets, demonstrating that the predicted peptide structure information is important for AMP prediction.

**Availability and implementation:**

A user-friendly webserver of sAMPpred-GAT can be accessed at http://bliulab.net/sAMPpred-GAT and the source code is available at https://github.com/HongWuL/sAMPpred-GAT/.

**Supplementary information:**

Supplementary data are available at *Bioinformatics* online.

## 1 Introduction

Due to the development of drug-resistant microbes, several alternative treatments have gained attention for treating multi-drug-resistant microbes caused by these diseases (Saxena and Gomber, 2010). Antimicrobial peptides (AMPs) as biomolecules have therapeutic functions. AMPs sequences have broad antibiotic-resistant activity against Gram-negative bacteria, cancer cells, fungi, etc. (Thomas *et al.*, 2010; Zasloff, 2002). Compared with the traditional antibiotic, AMPs interact with microbial membranes and penetration to promote the death of the target microbe and reduce the development of drug-resistant (Gaspar *et al.*, 2013; Wei *et al.*, 2021a,b). Therefore, AMPs identification and investigation are important for understanding the mechanism of new drug design (de la Fuente-Nunez *et al.*, 2017; Yan *et al.*, 2022a,b).

Several studies have been proposed to recognize and design new AMPs with different functional activities. AMPs are typically small proteins with a length of <100 amino acids, and have low homology with other peptide sequences (Bahar and Ren, 2013; Jenssen *et al.*, 2006). Identifying AMPs is a more challenging task. The computational methods developed to identify novel AMPs contain two parts, including databases construction and machine learning predictors (Barreto-Santamaria *et al.*, 2019; Pang *et al.*, 2021).

Many databases have been constructed to store the experimental validation AMP sequences. APD (Wang and Wang, 2004) is one of the earliest AMP databases, and APD3 (Wang *et al.*, 2016) is its third version. APD3 database contains 2747 sequences with annotated AMPs and their functionary activity. ADAM (Lee *et al.*, 2015) mainly concentrates on the relationship between AMP sequences and structures. SATPdb (Singh *et al.*, 2016) provides a large number of AMP structures, a majority of which are predicted by computational tools. Several high-throughput AMP databases have been proposed to evaluate the functionary activity and specific physicochemical information of the collection sequences, such as DRAMP 3.0 (Shi *et al.*, 2022), dbAMP 2.0 (Jhong *et al.*, 2022), etc.

Some machine learning methods have been developed for identifying different functional activities of AMP. These approaches can be divided into two categories based on the machine learning algorithm. First, the predictors constructed based on conventional methodologies. Among those methods, Support Vector Machine (SVM), Random Forest (RF), decision tree (DT) and ensemble learning methods are the widely used methods, such as TP-MV (Yan *et al.*, 2022a,b), iProt-Sub (Song *et al.*, 2019), etc. AVPpred (Thakur *et al.*, 2012) is the first attempt to predict the anti-virus peptides by integrating amino acid composition and physicochemical features with SVM. Second, the predictors utilize the deep learning framework to distinguish AMPs and non-AMPs. Veltri *et al.* (2018) utilized the deep neural network (DNN) architecture to identify AMPs. AMPlify (Li *et al.*, 2022) utilizes a bidirectional long short-term memory (Bi-LSTM) layer to distinguish AMPs from non-AMPs. In addition to the aforementioned machine learning methods, the appropriate peptide features are important factors to improve the prediction performance (Bhadra *et al.*, 2018). The sequence order, composition, physicochemical properties and structure features have been widely used to predict AMPs. For example, amino acid composition (AAC) and dipeptide composition are two widely used peptide descriptors (Guo *et al.*, 2021; Wei *et al.*, 2018). The composition, transition and distribution (CTD) feature and the pseudo amino acid composition (PseAAC) feature integrate the sequence property with the physical-chemical property (Bhadra *et al.*, 2018).

In the last decade, graph neural network (GNN) methods have been widely used in addressing many tasks in computational biology (Chen *et al.*, 2021; Gligorijević *et al.*, 2021). GNN methods are able to extract the task-specific features directly from the contact map or the protein sequence, overcoming the limitations of the handcrafted features. Graph convolutional network (GCN) is one of the most frequently used GNN methods. Most of protein function predictors based on the GCN construct the generalizing convolutional framework by utilizing the effectively graph-like molecular representations, and convert them into complex features by using multiple convolution layers. Compared with GCN treating all neighbor nodes equally, Graph Attention Network (GAT) aggregates the neighbor information based on the attention mechanism (Lai and Xu, 2022). GAT methods have been successful applied for predicting the protein structures (Wei, 2019), biochemical activity of drug identification (Zhao *et al.*, 2021), etc.

Previous studies have showed that the predicted structures of proteins are useful for protein function prediction (Xia *et al.*, 2021). The peptide structures can be accurately predicted by the computational methods only based on their peptide sequences (Jumper *et al.*, 2021; Yang *et al.*, 2020), providing an opportunity to improve the performance of AMP prediction by making full use of the predicted structure information. In this regard, we make an attempt to use the predicted peptide structures to predict the AMPs based on the GAT. The GAT framework captures the structure information and the spatial relationships among the residues along with the peptide. Experimental results show that the sAMPpred-GAT outperforms the other state-of-the-art methods in terms of AUC, and achieves better or highly comparable performance in terms of the other metrics on the eight independent test datasets. The hierarchical cluster analysis validated that the data-driven features learned by the sAMPpred-GAT are specific for AMP, which provided more information for further analysis. Furthermore, a user-friendly web server has been established at http://bliulab.net/sAMPpred-GAT.

## 2 Materials and methods

### 2.1 Independent test datasets

To objectively evaluate the performance of our sAMPpred-GAT and the other prediction methods, we use seven independent test AMP datasets from Xu *et al.* (2021), including XUAMP (Xu *et al.*, 2021), APD3 (Wang *et al.*, 2016; Xu *et al.*, 2021), DRAMP (Fan *et al.*, 2016; Kang *et al.*, 2019; Xu *et al.*, 2021), LAMP (Xu *et al.*, 2021; Ye *et al.*, 2020; Zhao *et al.*, 2013), CAMP (Thomas *et al.*, 2010; Waghu *et al.*, 2016; Xu *et al.*, 2021), dbAMP (Jhong *et al.*, 2019; Xu *et al.*, 2021) and YADAMP (Piotto *et al.*, 2012; Xu *et al.*, 2021). For detailed information of these seven datasets, please referred to Xu *et al.* (2021). Following the process employed by (Xu *et al.*, 2021), we construct an additional non-redundant independent test dataset named DBAASP, whose positive samples are extracted from an updated database (Pirtskhalava *et al.*, 2021) sharing <90% similarities, and the negative samples are downloaded from UniProt Consortium (2015) sharing <40% similarities.

**Fig. 1.**
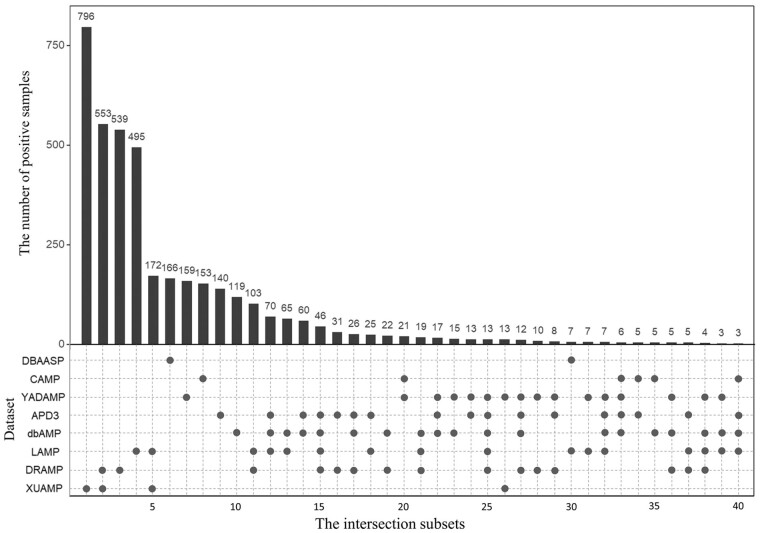
The number of overlapping positive samples among the eight independent test positive datasets. There are two parts in this figure. The upper part shows the number of positive samples in each intersection subset. Only the intersection subsets with more than two positive samples are shown. The bottom part shows the detailed information of the eight independent test positive datasets, where the black spots indicate that the corresponding independent test positive datasets listed in the *y*-axis share the intersection subset listed in the same column in the upper part. For examples, for column 11, there are 2 black spots in the bottom part, indicating that the independent test datasets LAMP and DRAMP share the intersection subset with 103 positive samples. In other words, LAMP and DRAMP share 103 common positive samples

The statistical information of all eight independent test datasets is listed in Table 1 and the relationship among these eight independent test datasets is shown in Figure 1 and Supplementary Figure S1. Figure 1 shows that for the eight independent test datasets, most of the positive samples are unique, and there are some overlapping positive samples among these independent datasets. The reason is that some recent independent datasets are constructed based on the previous ones. For example, XUAMP is constructed based on the DRAMP, LAMP, YADAMP, etc. (Xu *et al.*, 2021). These eight independent test datasets have unique positive samples, and many existing predictors are evaluated on them. Therefore, all these eight independent test datasets are selected to evaluate our method, and compare with the other existing methods. Supplementary Figure S1 shows that the sequence length distributions between positive and negative samples are similar in XUAMP and DRAMP, while the distributions are different in the other six independent test datasets. Furthermore, XUAMP and DRAMP are the top two largest test datasets (see Table 1). Therefore, XUAMP and DRAMP are used as the main test datasets to evaluate the performance of different methods. In order to comprehensively evaluate the performance of sAMPpred-GAT, it is also evaluated on the six test datasets with different distributions.

**Table 1.** Statistical information of the eight independent test datasets

Independent test datasets^a^	Positive	Negative
XUAMP	1536	1536
APD3	494	494
DRAMP	1408	1408
LAMP	1054	1054
CAMP	203	203
dbAMP	522	522
YADAMP	324	324
DBAASP	178	178

a The relationship among the eight independent test datasets is shown in Figure 1. We adopt CD-HIT (Huang *et al.*, 2010) to remove the redundancy between the independent datasets and the training sets. Following (Veltri *et al.*, 2018), the similarity between positive training samples and positive independent test samples is <90%, and the similarity between negative training samples and negative independent test samples is <40%.

### 2.2 Benchmark datasets

In this study, we construct a pretraining dataset, and based on which a benchmark dataset is constructed. The pretraining dataset is used for pretraining the proposed method to initialize the GAT framework, and the benchmark dataset is utilized for training and predicting the proposed method. The pretraining dataset containing 5536 AMPs and 5536 non-AMPs was constructed based on SATPdb (Singh *et al.*, 2016), ADAM (Lee *et al.*, 2015), AMPfun (Chung *et al.*, 2019), APD3 (Wang *et al.*, 2016), CAMP (Thomas *et al.*, 2010; Waghu *et al.*, 2016), LAMP (Ye *et al.*, 2020; Zhao *et al.*, 2013), DRAMP (Fan *et al.*, 2016), dbAMP (Jhong *et al.*, 2019) and UniProt Consortium (2015) databases. The sequence similarity between any two AMPs is <90%, and the sequence similarity between any two non-AMPs is <40% (Veltri *et al.*, 2018). Then, a benchmark dataset is constructed to avoid the misleading model prediction caused by the length distribution gap between AMPs and non-AMPs. We selected the positive samples and negative samples with the lengths in the range of 40–100 residues from the pretraining dataset to construct the benchmark dataset. The sequence length distributions of the samples in the benchmark dataset are shown in Supplementary Figure S2.

For model training, 80% of the samples in the benchmark dataset are randomly selected as the training dataset, and the remaining 20% are used as a validation dataset to optimize the parameters. The detailed steps of the dataset construction are described in Supplementary File S1.

### 2.3 Overview of sAMPpred-GAT

**Fig. 2.**
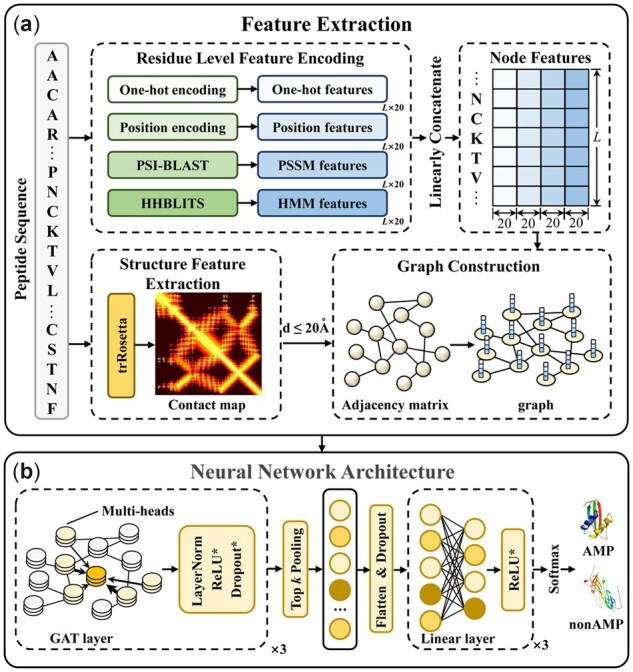
The framework of sAMPpred-GAT. (**a**) The flowchart of feature extraction and graph construction process. For the residue-level features, we extract four different features, including one-hot features, position encoding features, PSSM features and HMM features. For structure features, we extract the contact maps by using trRosetta (Yang *et al.*, 2020). The four residue-level features represented as the nodes and the adjacency matrix information represented as the edges are fed into the GAT framework. (**b**) The neural network architecture of sAMPpred-GAT. We use three GAT layers with multi-head attention to capture the information from the neighbors. The GAT module utilizes both the contact map information and four residue-level features as described above, and outputs the sequence-level features in the last layer. Then we utilize the top *k* pooling to transform the graphs into vectors with fixed length, which is the graph level representation. The linear layers are used to make the final prediction. The methods with suffix * indicate that they are not used in the last layer

The framework of sAMPpred-GAT is shown in Figure 2. The sAMPpred-GAT predictor extracts the residue-level features from the sequence information and the spatial relationships among the residues from the predicted structural information. Then the graphs are constructed to integrate the information of peptides, where the edges represent the structural information and the nodes represent the residue information, including sequence information and evolutionary information. Subsequently, we utilize the GAT to extract the features from the graphs-based data. Finally, we use the linear layer to predict whether a new peptide is an AMP or not.

### 2.4 Sequence feature extraction and graph construction module

In this section, we represent the sequences from two perspectives information, including residue-level features and structural features. Specifically, the residue-level features are represented by the amino acid composition information, amino acid position information and evolutionary information. The structural information is extracted by the trRosetta (Yang *et al.*, 2020), which is converted the raw sequences into the corresponding structures, which contain the distances and orientations between all the amino acid pairs along the sequence.

#### 2.4.1 Residual level features encoding

The proposed method utilizes four comprehensive features to represent residue-level features, including One-hot encoding features, Position encoding features, Position Specific Scoring Matrix (PSSM) features, and Hidden Markov Models (HMM) features.

One-hot encoding: to represent the composition information, we use one-hot to encode 20 standard amino acids. One-hot is a binary vector with only one position having a value of 1, representing the current acid amino, and the rest are set as 0. For example, amino acid A is coded as ${\left[1,0,0,\ldots ,0\right]}_{20}$. The dimension of the one-hot feature is $\left(L\times 20\right)$-D.

Position encoding: position encoding is claimed as an effective descriptor in many applications calculated by Vaswani *et al.* (2017):

(1) $$PE\left(\mathrm{pos},2i\right)=\sin\left(\frac{\mathrm{pos}}{b^{\frac{2i}{d}}}\right)$$

(2) $$PE\left(\mathrm{pos},2i+1\right)=\cos\left(\frac{\mathrm{pos}}{b^{\frac{2i}{d}}}\right)$$

where the pos indicates the position of the amino acid in the sequence (0≤pos≤L-1). L represents the length of the peptide sequence. The variable 2i and 2i+1 (0≤i<10) represent each position of the vector. It can be seen from Equations (1 and 2) that the values of odd positions and even positions are distinct. The b and *d* are constants, and their values are set to 1000 and 20 in this study, respectively. The dimension of the position encoding feature is $\left(L\times 20\right)$-D.

PSSM encoding: PSSM is one of the widely used evolutionary profiles, which is generated through the multiple sequence alignment (MSA) by running PSI-BLAST(Altschul *et al.*, 1997) (‘-num_ iterations 3 -evalue 0.01’). The query sequence is searched by the PSI-BLAST tool through the nrdb90 database (Holm and Sander, 1998). Because the length of AMP sequences is <100, the alignments of several peptides cannot be generated. For these peptides, their PSSMs are produced by the PSI-BLAST to search the NR database (O'Leary *et al.*, 2016) instead of the nrdb90 database (Holm and Sander, 1998). The dimension of the PSSM feature is $\left(L\times 20\right)$-D.

HMM encoding: in this study, the HMM profile is obtained by searching the query sequence against the Uniclust30 database using HHblits (Remmert *et al.*, 2012) with parameters ‘-n 3 -e 0.01’. The dimension of HMM is L×30, where the first 20 columns represent the amino acids match state frequencies, and the rest 10 columns represent seven translation frequencies and three local diversities. In this study, the first 20 columns are used as the residue-level features, which are similar to the PSSM profile (Yan *et al.*, 2019). According to the HHblits manual (Remmert *et al.*, 2012), $h_{ij}$ in the HMM profile is calculated by $-1000*\log_{2}{h^{\prime }}_{ij}$, where ${h^{\prime }}_{ij}$ denotes the amino acid match state frequency. Therefore, each score ${h^{\prime }}_{ij}$ is converted as (Yan *et al.*, 2019):

(3) $${h^{'}}_{ij}=2^{-0.001\times h_{ij}}(i\in \left[1,L\right],j\in \left[1,20\right])$$

Finally, the residue-level features are represented by integrating four descriptors, including one-hot encoding, position encoding, PSSM encoding and HMM encoding. The dimension of the residue-level feature is $\left(L\times 80\right)$-D.

#### 2.4.2 Structure feature encoding

The study shows that the AMP sequences have low sequence homology, but the corresponding structures of the AMPs may have high similarity (Wei *et al.*, 2021a,b). Therefore, the structural properties are able to effectively predict the AMPs and non-AMPs. Because the peptide structures validated by the experimental method are limited, their structures are predicted by computational methods, such as trRosetta (Yang *et al.*, 2020), etc.

The trRosetta predicts the protein structures based on the predicted contact maps. The predicted contact map contains distance information and angles of all residue pairs. In this study, we utilize the distance information between pairs of $C_{\beta }-C_{\beta }$ atoms in contact map to represent the spatial relationship of the residues. The distance information $A\in R^{n\times n}$ is a binary matrix, where n denotes the number of amino acids, and the element $A_{ij}$ is defined as (Gligorijević *et al.*, 2021):

(4) $$A_{u,t}=\left\{\begin{array}{c} 1,\;\mathrm{if}\;D_{u,t}\leq D_{\mathrm{th}}\;\mathrm{or}\;u=t\\ 0,\;\mathrm{otherwise} \end{array}\right.$$

where $D_{u,t}$ is the distance between atoms u and t, $D_{\mathrm{th}}$ is the threshold distance. In this study, $D_{\mathrm{th}}$ is set as 20 Å, which is optimized on the validation dataset. The input MSA of the peptide sequence is constructed by the HHblits, and the other parameters of trRosetta are set as default values.

#### 2.4.3 Graph construction

In this section, we utilize the graph to represent the peptide sequence, where the nodes consist of the residues-level information, and the edges are constructed according to the distance of the inter-residue pairs based on the structural information.

For discussion convenience, a graph is defined as $G=\left(V,E\right)$, where $V=\left\{v_{i}\right\}(i\in \left[1,n\right])$ is the set of nodes, and $v_{i}\in R^{d}$ is a node feature represented by the residue-level feature with the dimension of d. $E=\left\{e_{ij}|A_{ij}=1\right\}$ stands for the edge set of the graph.

### 2.5 Neural network architecture module

After constructing the graphs based on structural features and sequence features, a neural network based on GAT is designed to integrate information from the neighbor nodes and performs the prediction. It consists of three main modules, including (i) graph attention layers, (ii) top *k* pooling and (iii) output layers. The graph attention layers are utilized to extract the structural information from the graph of the sequence. The top *k* pooling can adaptively select the top *k* most informative nodes to generate the graph-level representation of the peptide. Finally, the output layers utilize the graph-level context vector to predict whether a peptide is an AMP or not.

#### 2.5.1 Graph attention layers

**Fig. 3.**
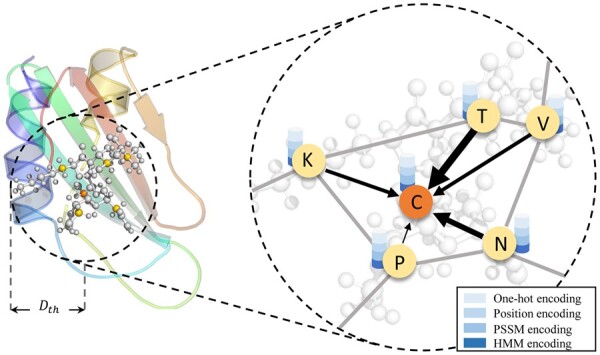
Visualization of converting the process AMP structure into a complex graph. The ball and stick model in the left subfigure represent the substructure of the peptide, where the center ball represents the central amino acid $C_{\beta }$ atom and the other balls are the $C_{\beta }$ atoms of amino acids within the threshold $C_{th}\;$predicted by trRosetta. The right subfigure represents the process of updating the central node features using GAT. The characters ‘K’, ‘T’, ‘V’, ‘N’, ‘P’ and ‘C’ represent the corresponding amino acids of the target $C_{\beta }\;$atoms

In this section, we utilize the GAT (Veličković *et al.*, 2017) to extract the structural information from the graph constructed based on the predicted structures. It leverages masked self-attention mechanism to automatically assign weights to neighbor nodes along the edges. The larger the weight is, the more important the neighbor node is. Therefore, GAT captures the local associations between amino acids and their neighbors. In addition, GAT is robust for predicted structure data. Due to a large gap between the real pairwise distance and the predicted pairwise distance caused by the structural prediction noise, GAT can adaptively assign smaller weights to those noisy data to reduce the negative impact of noisy data on the prediction results. The process is shown in Figure 3.

Let $h_{v}^{\left(t\right)}\in R^{d_{t}}$ denotes the representation of node v with a dimension $d_{t}$ in the layer $t\left(\;1\leq t\leq n_{l}\right)$. $h_{v}^{\left(0\right)}=x_{v}\in R^{d}$ is the feature of node v constructed in Section 2.4. $h_{v}^{n_{l}}$ is the feature of node v after the last GAT layer. The neighborhood of a node v is defined as $N\left(v\right)=\{u\in V|e_{uv}\in E\}$. GAT updates the node representation in an iterative way by using the following rule (Veličković *et al.*, 2017):

(5) $$h_{v}^{\left(t\right)}=\sum_{u\in N\left(v\right)\cup \left\{v\right\}}\alpha_{uv}^{\left(t\right)}W^{\left(t\right)}h_{u}^{\left(t-1\right)}$$

where $W^{\left(t\right)}\in R^{d_{t}\times d_{t-1}}$ is the projection transformation matrix in the layer t. Compared with the GCN utilizing the fixed weights based on the degrees of the respective nodes, GAT aggregates the neighboring features with the self-attention-based weights. The attention coefficients $\alpha_{uv}^{\left(t\right)}$ is computed by (Veličković *et al.*, 2017):

(6) $$\alpha_{uv}^{\left(t\right)}=\frac{\exp(g(a^{\left(t\right)T}[W^{\left(t\right)}h_{v}^{\left(t-1\right)}||W^{\left(t\right)}h_{u}^{\left(t-1\right)}]))}{\sum_{k\in N\left(v\right)\cup \left\{v\right\}}\exp(g(a^{\left(t\right)T}[W^{\left(t\right)}h_{v}^{\left(t-1\right)}||W^{\left(t\right)}h_{k}^{\left(t-1\right)}]))}$$

where g(·) is a LeakyReLU activation function and $a^{\left(t\right)}\in R^{{2d}_{t}}$ is a vector of learnable parameters. ∥ represents concatenation.

Besides, multi-head attention mechanism (Vaswani *et al.*, 2017) is used in this study for the polysemous phenomenon. GAT with H heads is equivalent to integrate *H* single GAT layers in parallel. Moreover, except for the last layer, ReLU (Nair and Hinton, 2010) and dropout (Srivastava *et al.*, 2014) are used after each GAT layer to enhance the ability of non-linear learning and generalization, respectively.

#### 2.5.2 Top *k* pooling

Top *k* pooling is an module to adaptively select a certain number of nodes (Gao and Ji, 2019). In this task, it converts the variable length amino acid sequence features into a fixed-length vector by selecting the top *k* residues (nodes) with the highest score.

Top *k* pooling computes the scalar projection score $s_{v}$ for a node v with feature $h_{v}$, which measures how much information of node v reserves when projected onto the direction of a learnable vector p. Then the new representation $h_{v}^{\prime }$ is calculated based on the indices corresponding to the top *k* nodes. The calculation process is as follows (Gao and Ji, 2019):

(7) $$s_{v}=\frac{h_{v}^{T}p}{∥p{∥}_{2}},\;v\in V$$

(8) $$\mathrm{idx}=\mathrm{to}p_{k}\left(s\right)$$

(9) $$h_{t}^{'}=h_{t}⊙\tanh\left(s_{t}\right),\;t\in \mathrm{idx}$$

where $s=\{s_{v}\in R|v\in V\}$ is the vector set of projection score; $\mathrm{to}p_{k}(\cdot )$ is used to select the top *k* most informative nodes and returns their indices set idx. tanh⁡(·) is a non-linear function and ⊙ is the elementwise product.

Finally, we obtain the graph representation z by concatenating them linearly:

(10) $$z=\;{∥}_{t\in \mathrm{idx}}h_{t}^{'}$$

#### 2.5.3 Output layers

To predict whether the input sequence is an AMP or not, the linear layers are needed to learn a more discriminative representation, defined as (Goodfellow *et al.*, 2016):

(11) $$x^{\left(t\right)}=W_{l}^{\left(t\right)}x^{\left(t-1\right)}+b^{\left(t\right)}$$

where $x^{\left(t-1\right)}$ and $x^{\left(t\right)}$ are the input vector and output vector in the tth linear layer, respectively. $x^{\left(0\right)}=\mathrm{flatten}(z)$. $W_{l}^{\left(t\right)}$ denotes the weight matrix and $b^{\left(t\right)}$ is the bias of tth linear layer. ReLU and dropout are used following each linear layer except the last one. After the last layer, a softmax function (Goodfellow *et al.*, 2016) is added to perform the final prediction.

(12) $$y_{i}=\frac{\exp\left(x_{i}^{\left(M\right)}\right)}{\sum_{j\in c}\exp\left(x_{j}^{\left(M\right)}\right)}$$

where c={0, 1} denotes the binary label. Here AMP is labeled as 1, while non-AMP is labeled as 0. $y_{i}\in \{y_{0},\;y_{1}\}$ denotes the negative or positive probabilistic. M is the total number of linear layers.

#### 2.5.4 The model implementation

We utilized the PyTorch (https://pytorch.org/) and PyTorch Geometric (Fey and Lenssen, 2019) to implement sAMPpred-GAT. The negative log-likelihood loss function is used to measure the gap between predicted labels and truth labels. The ADAM algorithm (Kingma and Ba, 2014) is adopted to optimize GAT with a batch size of 512 during the training process. The initial learning rate of pretraining is set as 0.001, which is decayed to 95% for every 5 epochs. A total of 50 epochs are iterated. Then a smaller learning rate of 0.0001 and fewer epochs 20 are adapted to train the model. The other parameters are the same in both the two stages. The hyperparameters are optimized based on the grid search strategy according to the maximum AUC. Please refer to Supplementary Tables S1 and S2 for more information.

### 2.6 Evaluation metrics

In this study, we utilize five metrics to evaluate the proposed method, which are calculated by:

(13) $$\left\{\begin{array}{c} A\mathrm{CC}=\;\frac{TP+TN}{TP+TN+FN+FP}\\ M\mathrm{CC}=\;\frac{TP\times TN-FP\times FN}{\sqrt{\left(TP+FN\right)\left(TP+FP\right)\left(TN+FP\right)\left(TN+FN\right)}}\\ \begin{array}{c} Sn=\;\frac{TP}{TP+FN}\\ Sp=\;\frac{TN}{TN+FP}\\ A\mathrm{UC}:\;\mathrm{Area}\;\mathrm{under}\;\mathrm{the}\;\mathrm{ROC}\;\mathrm{Curve}\; \end{array} \end{array}\right.$$

where *TP*, *FP*, *TN* and *FN* are the number of true positives, false positives, true negatives and false negatives, respectively (Charoenkwan *et al.*, 2021; Chen and Shi, 2019; Jin *et al.*, 2020).

## 3 Results and discussion

### 3.1 Performance on XUAMP independent test dataset

In this section, we compare the proposed method with nine state-of-the-art methods on the independent test XUAMP dataset, including amPEPpy (Lawrence *et al.*, 2021), AMPfun (Chung *et al.*, 2019), AMPEP (Bhadra *et al.*, 2018), ADAM-HMM (Lee *et al.*, 2015), ampir (Fingerhut *et al.*, 2021), AMPScannerV2 (Veltri *et al.*, 2018), AMPGram (Burdukiewicz *et al.*, 2020), Deep-AMPEP30 (Yan *et al.*, 2020) and CAMP-ANN (Waghu *et al.*, 2016). For the sake of avoiding overestimating the performance of sAMPpred-GAT, the positive samples in the benchmark dataset sharing more than 90% similarities with any positive sample in the XUAMP independent test dataset are removed following (Veltri *et al.*, 2018). The negative samples in the benchmark dataset sharing more than 40% similarities with any negative sample in the XUAMP independent test dataset are removed as well. To avoid the randomness of the initialization parameters, the proposed method sAMPpred-GAT is run 10 times with random seeds.

**Fig. 4.**
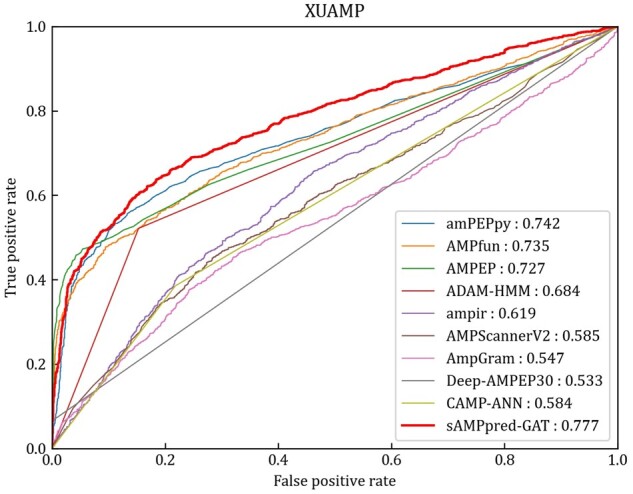
ROC curves of sAMPpred-GAT and the other state-of-the-art methods on the XUAMP test dataset. The results of the other nine predictors are from Xu *et al.* (2021)

The experimental results of different methods are shown in Figure 4 and Table 2, from which we can observe that sAMPpred-GAT achieves the best performance in terms of AUC, ACC, MCC and Sn, and achieves highly comparable Sp. The results show that the proposed method achieves the highest AUC with fewer false positives. Therefore, sAMPpred-GAT is a useful predictor for identifying AMPs.

**Table 2.** The performance of sAMPpred-GAT and the other existing predictors on the independent test XUAMP dataset in terms of ACC, MCC, Sn and Sp^a^

Method	ACC	MCC	Sn	Sp	Source
amPEPpy	0.679	0.431	0.400	0.958	Xu *et al.* (2021)
AMPfun	0.674	0.414	0.406	0.943	Xu *et al.* (2021)
AMPEP	0.661	0.429	0.330	0.992	Xu *et al.* (2021)
ADAM-HMM	0.684	0.390	0.521	0.847	Xu *et al.* (2021)
Ampir	0.563	0.156	0.266	0.859	Xu *et al.* (2021)
AMPScannerV2	0.568	0.137	0.523	0.613	Xu *et al.* (2021)
AmpGram	0.564	0.131	0.445	0.682	Xu *et al.* (2021)
Deep-AMPEP30	0.533	0.183	0.065	**1.0**	Xu *et al.* (2021)
CAMP-ANN	0.584	0.182	0.385	0.782	Xu *et al.* (2021)
sAMPpred-GAT^b^	**0.715 ± 0.01**	**0.464 ± 0.011**	**0.530 ± 0.038**	0.9 ± 0.02	This study

a The ACC, MCC, Sn and Sp values of nine compared methods are calculated based on the detailed prediction results of these predictors reported in Xu *et al.* (2021), which can be downloaded from http://bliulab.net/sAMPpred-GAT/data/.

b The reported results of the proposed method are the average and standard deviation after performing the randomness initialization parameters 10 times.

*Note*: The best performance of each metric is highlighted in bold.

### 3.2 Performance on the other independent test datasets

In this section, we comprehensively evaluate the performance of sAMPpred-GAT on the seven independent test datasets, including APD3 (Wang *et al.*, 2016; Xu *et al.*, 2021), DRAMP (Fan *et al.*, 2016; Kang *et al.*, 2019; Xu *et al.*, 2021), LAMP (Xu *et al.*, 2021; Ye *et al.*, 2020; Zhao *et al.*, 2013), CAMP (Thomas *et al.*, 2010; Waghu *et al.*, 2016; Xu *et al.*, 2021), dbAMP (Jhong *et al.*, 2019; Xu *et al.*, 2021), YADAMP (Piotto *et al.*, 2012; Xu *et al.*, 2021) and DBAASP.

For the sake of avoiding overestimating the performance of sAMPpred-GAT, for each target independent test dataset, the positive samples in the benchmark dataset sharing more than 90% similarities with any positive sample in the target independent test dataset are removed following (Veltri *et al.*, 2018). The negative samples in the benchmark dataset sharing more than 40% similarities with any negative sample in the target independent test dataset are removed (Veltri *et al.*, 2018).

**Fig. 5.**
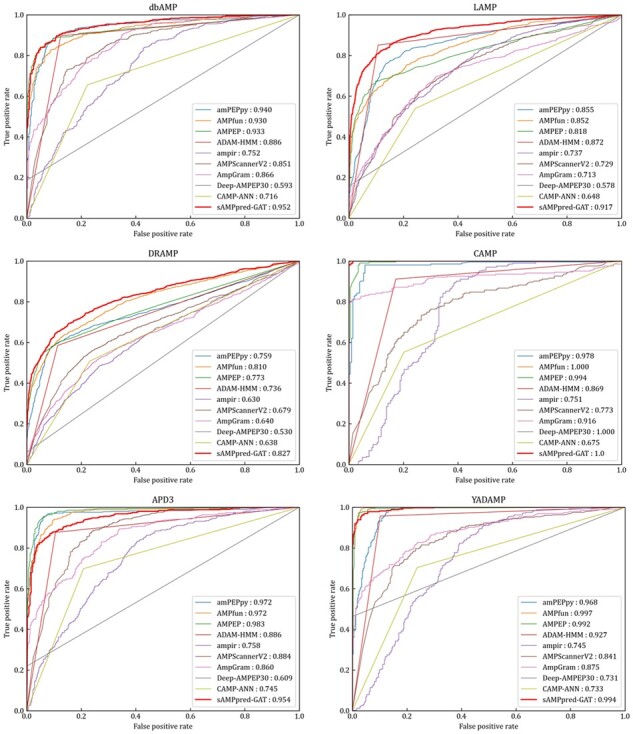
ROC curves of sAMPpred-GAT and the other nine predictors on the six independent test datasets. The results of the other nine predictors are from Xu *et al.* (2021)

We evaluate the performance of sAMPpred-GAT and the other state-of-the-art methods on the following independent test datasets: APD3 (Wang *et al.*, 2016), DRAMP (Fan *et al.*, 2016; Kang *et al.*, 2019), LAMP (Ye *et al.*, 2020; Zhao *et al.*, 2013), CAMP (Thomas *et al.*, 2010; Waghu *et al.*, 2016), dbAMP (Jhong *et al.*, 2019) and YADAMP (Piotto *et al.*, 2012). The results are listed in Figure 5, Supplementary Tables S3 and S4. Although sAMPpred-GAT outperforms most of the compared methods, it achieves similar performance as amPEPpy (Lawrence *et al.*, 2021), AMPfun (Chung *et al.*, 2019) and AMPEP (Bhadra *et al.*, 2018) on these six independent test datasets. However, we found that many samples in the six independent test datasets already exist in the corresponding training sets of amPEPpy, AMPfun and AMPEP (see Supplementary Table S5). Therefore, the performance of these three methods on the six independent test datasets would be overestimated. In order to validate this point, we removed all these overlapping samples from the six independent test datasets to make sure that there is no overlapping sample between the training sets of the three compared methods and the six independent test datasets, and then the sAMPpred-GAT, amPEPpy, AMPfun and AMPEP were re-evaluated on these six non-redundant independent test datasets (see Supplementary Tables S6–S8). From these tables, we can see that the performance of amPEPpy, AMPfun and AMPEP decreases obviously, and sAMPpred-GAT outperforms all these three compared methods. Therefore, we conclude that the performance of amPEPpy, AMPfun and AMPEP on the six independent test datasets is indeed overestimated, and sAMPpred-GAT outperforms all the compared methods. Moreover, we further evaluate the performance of sAMPpred-GAT on the independent test dataset DBAASP, and its performance is compared with amPEPpy, AMPfun and AMPEP (see Supplementary Fig. S3). The results further confirm the better of sAMPpred-GAT.

The sAMPpred-GAT predictor captures more discriminative features, while most of the existing methods utilized the hand-crafted features based on the experience. Therefore, the sAMPpred-GAT method is a useful tool for AMPs prediction.

### 3.3 Effectiveness of structural features

In this section, we analyze the effectiveness of structural features and the different numbers of GAT layers on the performance of sAMPpred-GAT on XUAMP dataset (see Table 3). The results show that when the number of GAT layers is set as three, which obtains the best performance in terms of AUC. The performance of sAMPpred-GAT decreases when the layer number is higher than three, caused by the problem of over-smoothing in GNNs (Chen *et al.*, 2020). Moreover, when the number of GAT layers is 0, we only utilize the residue-level sequence features. The corresponding performance of sAMPpred-GAT is low, indicating that the structural features are discriminative and can improve the predictive performance. Therefore, the proposed method utilizes the GAT framework to extract the discriminative features from the structural properties of peptides so as to improve the predictive performance.

**Table 3.** The predictive performance of sAMPpred-GAT with different GAT layers on independent test XUAMP dataset

#Layers	AUC	ACC	MCC	Sn	Sp
0	0.671	0.627	0.258	0.547	0.707
1	0.737	0.684	0.392	0.517	0.852
2	0.767	0.709	0.440	**0.551**	0.866
**3**	**0.777**	**0.715**	**0.464**	0.530	**0.9**
4	0.772	0.711	0.451	0.538	0.884

*Note*: The best performance of each metric is highlighted in bold.

### 3.4 Analysis of sequential features

In this section, we conduct an ablation experiment to evaluate the contribution of different residue-level sequential features to the performance of the sAMPpred-GAT predictor on the independent test XUAMP dataset. The results are shown in Table 4, from which we see that when residue-level features with different properties are linearly combined, the performance of sAMPpred-GAT improves steadily. When we utilize the position encoding residue-level features, the performance of the proposed method improves obviously. Specifically, the position features improve the predictive performance by 4.3% and 4.5% in terms of AUC and ACC, respectively. The reason is that the machine learning predictors based on the GNN fail to capture the position information (Liu *et al.*, 2020; You *et al.*, 2019). As a result, the features associated with the position information is able to improve the prediction performance of sAMPpred-GAT. In addition, the proposed method extracts the evolutionary information by using neural networks, which can better distinguish the AMPs. Therefore, sAMPpred-GAT accurately predicts the AMPs by integrating the position and evolution information via using the GAT.

**Table 4.** The influence of different features and their combinations on the performance of sAMPpred-GAT evaluated on the XUAMP dataset

Features	AUC	ACC	MCC	Sn	Sp
One-hot	0.705	0.650	0.323	0.470	0.831
One-hot + position	0.748	0.695	0.416	0.523	0.867
One-hot + position + PSSM	0.766	0.709	0.452	0.518	0.899
One-hot + position + PSSM + HMM	**0.777**	**0.715**	**0.464**	**0.530**	**0.9**

*Note*: The best performance of each metric is highlighted in bold.

### 3.5 Visualization of the features extracted by sAMPpred-GAT

**Fig. 6.**
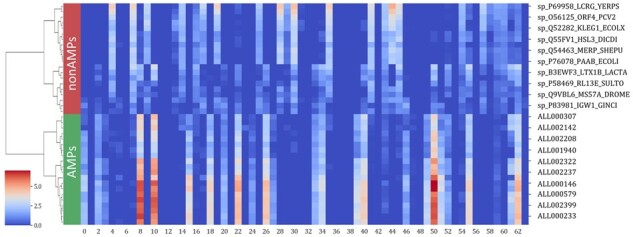
Analysis of the learned features extracted by the sAMPpred-GAT by running agglomerative clustering over 40 peptides from XUAMP dataset. The learned features are extracted from the penultimate full connection layer with 64-D

The sAMPpred-GAT predictor mainly utilizes the GAT framework to automatically learn the inherent features from the structural information, sequence information and evolutionary information. To intuitively demonstrate the effectiveness of the learned features, we utilize the agglomerative clustering algorithm (Rokach and Maimon, 2005) to hierarchically cluster the learned features of the peptides from the XUAMP dataset. The results are shown in Figure 6, from which we can observe that: (i) the learned features with high values form the blocks, which indicates that peptides with similar functions have similar characteristics, and the peptides with different functions have different features. (ii) The peptides from the same function were groups into the same cluster. The learned features are more clearly to distinguish the AMP and non-AMP in feature space distribution. Therefore, the learned features extracted by sAMPpred-GAT are discriminative, contributing to the performance of sAMPpred-GAT for predicting AMPs.

## 4 Conclusion

AMPs are essential biomolecules of therapeutic peptides for the immune system of organisms. In this study, we proposed a sAMPpred-GAT predictor based on the predicted peptide structure information for AMPs recognition. The proposed method utilizes structural information, evolutionary profiles and sequence features to construct the graphs. Then the proposed method extracts the discriminative features from the graph by using the GAT framework. Finally, the optimized features are fed into the output layer to predict the AMPs. Experimental results show that the sAMPpred-GAT outperforms the other state-of-the-art methods in terms of AUC, and achieves better or highly comparable performance in terms of the other metrics on the eight independent test datasets. The framework using structural features and GAT layers can learn the inherent features with more discriminative power so as to improve the predictive performance. The GAT would have many other potential applications in bioinformatics, such as non-coding RNA and disease association identification, peptide prediction, etc.

## Supplementary Material

btac715_Supplementary_Data
