# Supplementary material for: sAMPpred-GAT: prediction of antimicrobial peptide by graph attention network and predicted peptide structure
Source: Bioinformatics. 2022 Nov 7;39(1):btac715. doi: 10.1093/bioinformatics/btac715 (PMC9805557; doi:10.1093/bioinformatics/btac715)
Supplement: btac715_Supplementary_Data [file btac715_supplementary_data.zip › btac715_Supplementary_Data/supplementary material.docx]

**Supplementary file.**

**S1. The construction of Benchmark datasets**

For the positive subset, we integrated eight AMP datasets, including SATPdb [1], ADAM [2], AMPfun [3], APD3 [4], CAMP [5, 6], LAMP [7, 8], DRAMP [9] and dbAMP [10]. We removed the non-standard residues and collected the sequences with length in a range of 10~100. Furthermore, to remove the sequence homology bias and redundancy, the sequence similarity in the same subset or with the independent test positive dataset XUAMP [11] was reduced to 90% [12] by using CD-HIT [13].

For the negative subset, we selected the non-AMP sequences from the UniProt database [14] without the keywords ‘antimicrobial’, ‘antibacterial’, ‘antifungal’, ‘anticancer’, ‘antiviral’, ‘antiparasitic’, ‘antibiotic’, ‘antibiofilm’, ‘effector’, ‘excreted’, ‘anti-microbial’, ‘anti-bacterial’, ‘anti-fungal’, ‘anti-cancer’, ‘anti-viral’, ‘anti-parasitic’ or ‘anti-biofilm’. In order to keep the negative samples sharing similar length distribution as that of the positive samples, we selected the negative samples with length in the range of 10 ~ 100 residues, and eliminated the non-standard residues. The sequence similarity in the same subset or in the negative dataset of the independent test dataset XUAMP [11] was reduced to 40% [12] by using CD-HIT [13]. We selected 5536 positive samples and 5536 negative samples to construct the pretraining dataset.

Next, a benchmark dataset is constructed to avoid the misleading model prediction caused by the length distribution gap between AMPs and non-AMPs. We selected the positive samples and negative samples with the lengths in the range of 40 ~ 100 residues from the pretraining dataset to construct the benchmark dataset (see **Fig. S2**).

When evaluating the performance of sAMPpred-GAT on the other seven independent test datasets, the positive samples in the benchmark dataset sharing more than 90% similarities with any positive sample in the corresponding independent test datasets and the negative samples in the benchmark dataset sharing more than 40% similarities with any negative sample in the corresponding independent test datasets are removed so as to fairly evaluate the performance of the proposed method.

**Table S1**. The hyper-parameter search ranges and the optimized values of sAMPpred-GAT.

| Modules | Parameters | Search range | Final |
| --- | --- | --- | --- |
| Feature extraction | D_th_ | [5, 10, 15, 20] | 20 |
| Network | #Layers of GAT | [1, 2, 3, 4] | 3 |
|  | Dropout rate | [0.3, 0.5, 0.7] | 0.5 |
|  | Hidden size | [64, 128] | 64 |
| Other parameters | Learning rate | [0.01, 0.001, 0.0001] | 0.0001 |

**Table S2**. The neural network parameters of sAMPpred-GAT.

| Modules | | Parameters |
| --- | --- | --- |
| Graph attention layers | GAT 1 | 1. GATConv (in_channels = 80, out_channels = 64, heads=8, concat=True) 2. LayerNorm (in_channels = 8 * 64) 3. ReLU () 4. Dropout (p = 0.5) |
|  | GAT 2 | 1. GATConv (in_channels = 8 * 64, out_channels = 64, heads=8, concat=True) 2. LayerNorm (in_channels = 8 * 64) 3. ReLU () 4. Dropout (p = 0.5) |
|  | GAT 3 | a) GATConv (in_channels = 8 * 64, out_channels = 64, heads=8, concat=False)  b) LayerNorm (in_channels = 64) |
| Top k pooling | | 1. TopKPooling (in_channels=64, ratio=10) 2. Flatten () 3. Dropout (p = 0.5) |
| Output layers | Linear 1 | 1. Linear (in_features = 10 * 64,   out_features = 64, bias = True)   1. ReLU () |
|  | Linear 2 | 1. Linear (in_features = 64,   out_features = 64, bias = True)  b) ReLU () |
|  | Linear 3 | 1. Linear (in_features = 64, out_features = 2, bias = True) |

**Table S3**. ACC values of sAMPpred-GAT and the other 9 predictors on six test datasets.

| Method | APD3 | DRAMP | LAMP | CAMP | dbAMP | YADAMP |
| --- | --- | --- | --- | --- | --- | --- |
| sAMPpred-GAT | 0.896 | 0.760 | 0.840 | 0.956 | 0.888 | 0.955 |
| amPEPpy | 0.939 | 0.734 | 0.765 | 0.948 | 0.889 | 0.915 |
| AMPfun | 0.916 | 0.739 | 0.759 | 0.968 | 0.863 | 0.954 |
| AMPEP | 0.936 | 0.712 | 0.755 | 0.973 | 0.766 | 0.969 |
| ADAM-HMM | 0.886 | 0.736 | 0.872 | 0.869 | 0.886 | 0.927 |
| ampir | 0.607 | 0.577 | 0.614 | 0.5 | 0.624 | 0.566 |
| AMPScannerV2 | 0.799 | 0.646 | 0.677 | 0.717 | 0.769 | 0.761 |
| AmpGram | 0.768 | 0.62 | 0.683 | 0.805 | 0.766 | 0.778 |
| Deep-AMPEP30 | 0.609 | 0.53 | 0.578 | 1.0 | 0.593 | 0.731 |
| CAMP-ANN | 0.745 | 0.638 | 0.648 | 0.675 | 0.716 | 0.733 |

**Table S4.** MCC values of sAMPpred-GAT and the other 9 predictors on six test datasets.

| Method | APD3 | DRAMP | LAMP | CAMP | dbAMP | YADAMP |
| --- | --- | --- | --- | --- | --- | --- |
| sAMPpred-GAT | 0.793 | 0.557 | 0.694 | 0.916 | 0.780 | 0.912 |
| amPEPpy | 0.879 | 0.506 | 0.557 | 0.897 | 0.78 | 0.83 |
| AMPfun | 0.832 | 0.511 | 0.546 | 0.938 | 0.73 | 0.911 |
| AMPEP | 0.873 | 0.495 | 0.558 | 0.946 | 0.779 | 0.938 |
| ADAM-HMM | 0.771 | 0.495 | 0.744 | 0.742 | 0.772 | 0.856 |
| ampir | 0.272 | 0.197 | 0.279 | 0 | 0.29 | 0.171 |
| AMPScannerV2 | 0.622 | 0.292 | 0.354 | 0.437 | 0.551 | 0.535 |
| AmpGram | 0.548 | 0.243 | 0.365 | 0.626 | 0.544 | 0.566 |
| Deep-AMPEP30 | 0.35 | 0.175 | 0.291 | 1.0 | 0.317 | 0.549 |
| CAMP-ANN | 0.492 | 0.287 | 0.303 | 0.361 | 0.436 | 0.467 |

**Table S5.** The proportion of overlapping positive samples in the independent test datasets, which are also included in the training datasets of the compared methods.

| Method | APD3 | DRAMP | LAMP | CAMP | dbAMP | YADAMP |
| --- | --- | --- | --- | --- | --- | --- |
| amPEPpy | 64.2% | 16.1% | 17.0% | 89.2% | 57.9% | 65.1% |
| AMPfun | 50.2% | 21.7% | 19.4% | 38.9% | 43.1% | 32.4% |
| AMPEP | 64.8% | 16.3% | 17.3% | 89.2% | 59.4% | 65.7% |

**Table S6.** AUC values of sAMPpred-GAT and the other 3 top performing predictors on six non-redundant independent test datasets.

| Method | APD3 | DRAMP | LAMP | CAMP | dbAMP | YADAMP |
| --- | --- | --- | --- | --- | --- | --- |
| sAMPpred-GAT | 0.955 | 0.828 | 0.925 | 1.0 | 0.954 | 0.993 |
| amPEPpy | 0.928 | 0.721 | 0.836 | 0.851 | 0.88 | 0.954 |
| AMPfun | 0.955 | 0.771 | 0.812 | 1.0 | 0.892 | 0.999 |
| AMPEP | 0.964 | 0.74 | 0.827 | 0.986 | 0.889 | 0.997 |

**Table S7.** ACC values of sAMPpred-GAT and the other 3 top performing predictors on six non-redundant independent test datasets.

| Method | APD3 | DRAMP | LAMP | CAMP | dbAMP | YADAMP |
| --- | --- | --- | --- | --- | --- | --- |
| sAMPpred-GAT | 0.896 | 0.760 | 0.840 | 0.956 | 0.888 | 0.955 |
| amPEPpy | 0.890 | 0.692 | 0.735 | 0.818 | 0.798 | 0.889 |
| AMPfun | 0.888 | 0.687 | 0.709 | 0.956 | 0.800 | 0.957 |
| AMPEP | 0.885 | 0.656 | 0.715 | 0.932 | 0.906 | 0.968 |

**Table S8.** MCC values of sAMPpred-GAT and the other 3 top performing predictors on six non-redundant independent test datasets.

| Method | APD3 | DRAMP | LAMP | CAMP | dbAMP | YADAMP |
| --- | --- | --- | --- | --- | --- | --- |
| sAMPpred-GAT | 0.793 | 0.557 | 0.694 | 0.916 | 0.780 | 0.912 |
| amPEPpy | 0.781 | 0.432 | 0.513 | 0.639 | 0.614 | 0.781 |
| AMPfun | 0.778 | 0.425 | 0.457 | 0.915 | 0.619 | 0.917 |
| AMPEP | 0.782 | 0.403 | 0.498 | 0.865 | 0.601 | 0.937 |


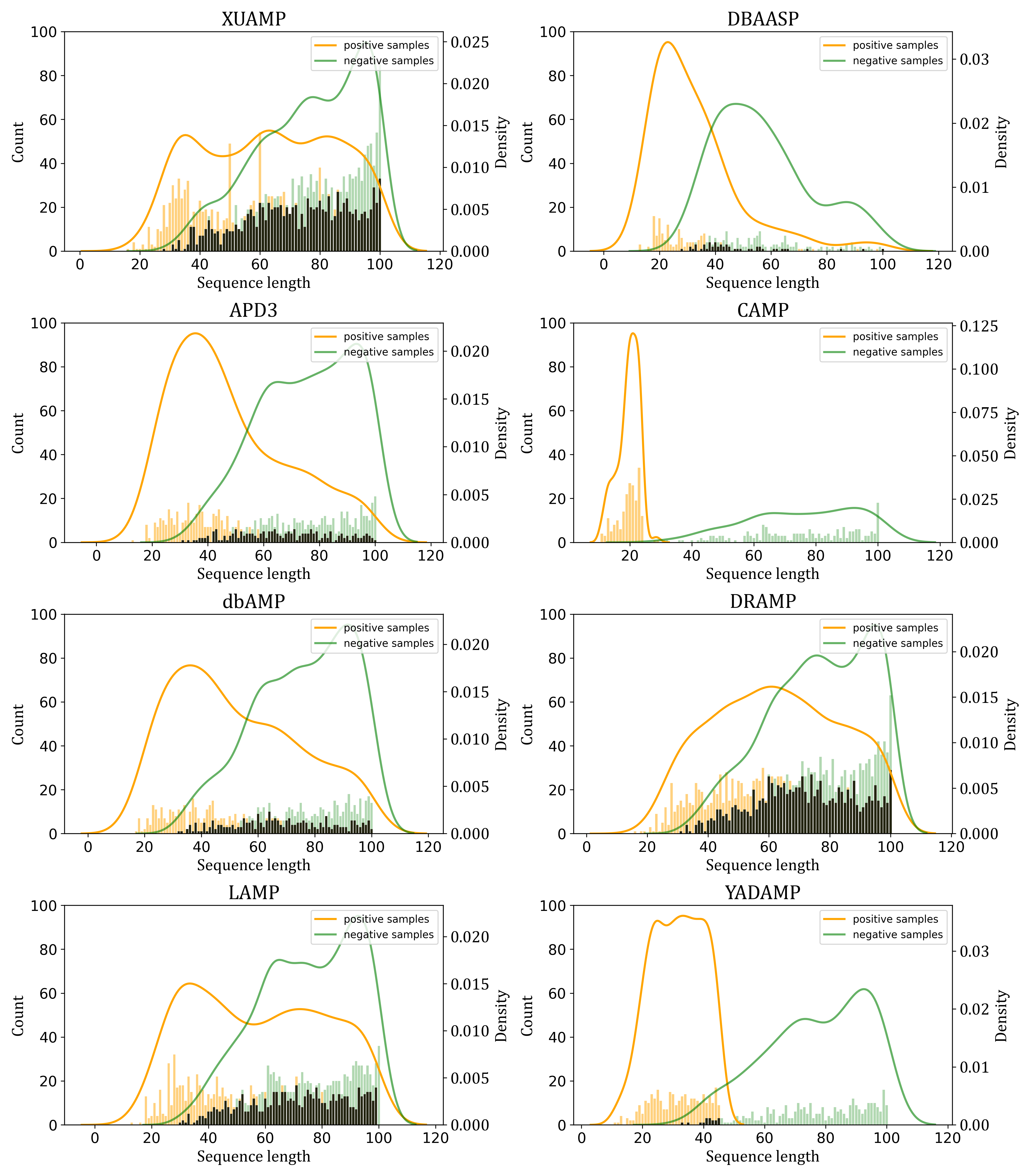


**Fig. S1**. The distributions of sequence lengths in independent test datasets, where the distributions of the positive samples are shown in yellow and negative samples are shown in green. The distribution of the overlap between positive samples and negative samples are shown in black.


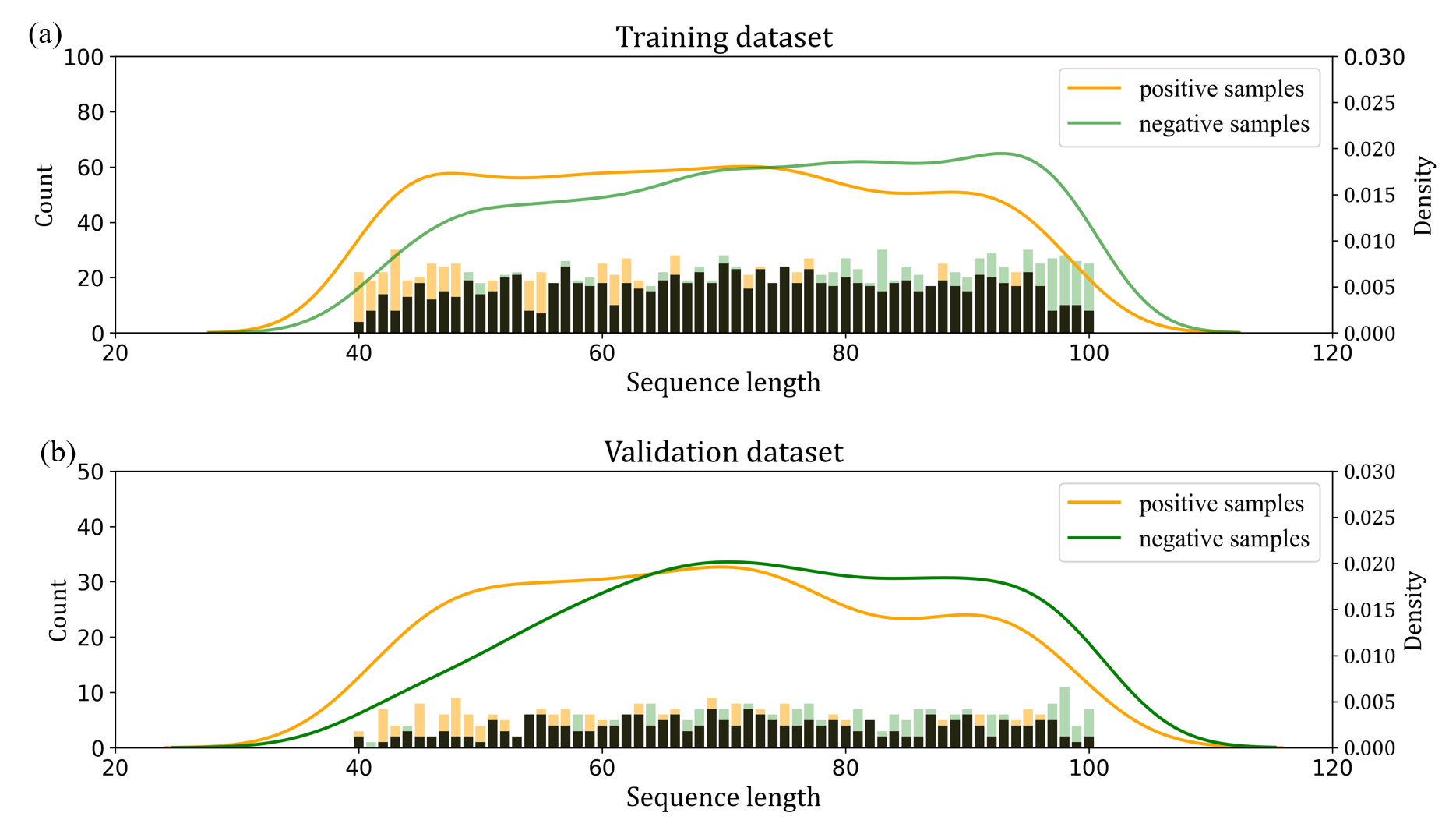


**Fig. S2**. The distributions of sequence lengths in training dataset (a) and validation dataset (b) of the benchmark dataset, where the distributions of the positive samples and negative samples are shown in yellow and green respectively, and the distributions of the overlap between positive samples and negative samples are shown in black.


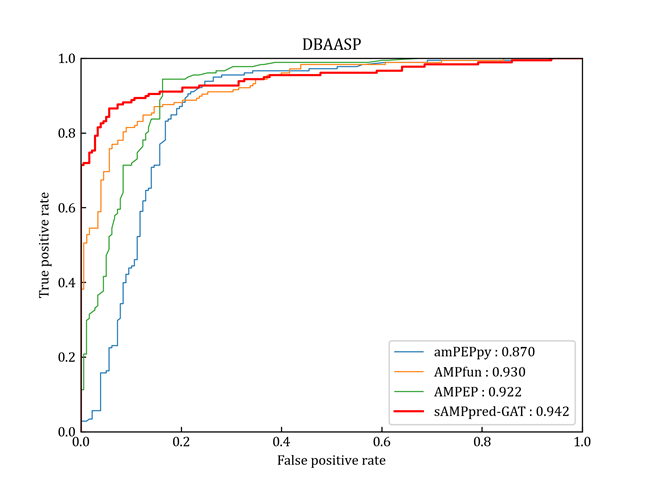


**Fig. S3.** ROC curves of sAMPpred-GAT, amPEPpy, AMPfun and AMPEP on the independent test dataset DBAASP.

**References**

1. Singh, S., et al., *SATPdb: a database of structurally annotated therapeutic peptides.* Nucleic Acids Res, 2016. **44**(D1): p. D1119-26.

2. Lee, H.T., et al., *A large-scale structural classification of antimicrobial peptides.* Biomed Res Int, 2015. **2015**: p. 475062.

3. Chung, C.R., et al., *Characterization and identification of antimicrobial peptides with different functional activities.* Brief Bioinform, 2019.

4. Wang, G., X. Li, and Z. Wang, *APD3: the antimicrobial peptide database as a tool for research and education.* Nucleic Acids Res, 2016. **44**(D1): p. D1087-93.

5. Thomas, S., et al., *CAMP: a useful resource for research on antimicrobial peptides.* Nucleic Acids Res, 2010. **38**(Database issue): p. D774-80.

6. Waghu, F.H., et al., *CAMPR3: a database on sequences, structures and signatures of antimicrobial peptides.* Nucleic Acids Res, 2016. **44**(D1): p. D1094-7.

7. Ye, G., et al., *LAMP2: a major update of the database linking antimicrobial peptides.* Database (Oxford), 2020. **2020**.

8. Zhao, X., et al., *LAMP: A Database Linking Antimicrobial Peptides.* PLoS One, 2013. **8**(6): p. e66557.

9. Fan, L., et al., *DRAMP: a comprehensive data repository of antimicrobial peptides.* Sci Rep, 2016. **6**: p. 24482.

10. Jhong, J.H., et al., *dbAMP: an integrated resource for exploring antimicrobial peptides with functional activities and physicochemical properties on transcriptome and proteome data.* Nucleic Acids Res, 2019. **47**(D1): p. D285-D297.

11. Xu, J., et al., *Comprehensive assessment of machine learning-based methods for predicting antimicrobial peptides.* Brief Bioinform, 2021. **22**(5).

12. Veltri, D., U. Kamath, and A. Shehu, *Deep learning improves antimicrobial peptide recognition.* Bioinformatics, 2018. **34**(16): p. 2740-2747.

13. Huang, Y., et al., *CD-HIT Suite: a web server for clustering and comparing biological sequences.* Bioinformatics, 2010. **26**(5): p. 680-2.

14. UniProt, C., *UniProt: a hub for protein information.* Nucleic Acids Research, 2015. **43**(D1): p. D204-D212.
