# Supplementary figures and images for: sAMPpred-GAT: prediction of antimicrobial peptide by graph attention network and predicted peptide structure
Source: Bioinformatics. 2022 Nov 7;39(1):btac715. doi: 10.1093/bioinformatics/btac715 (PMC9805557; doi:10.1093/bioinformatics/btac715)

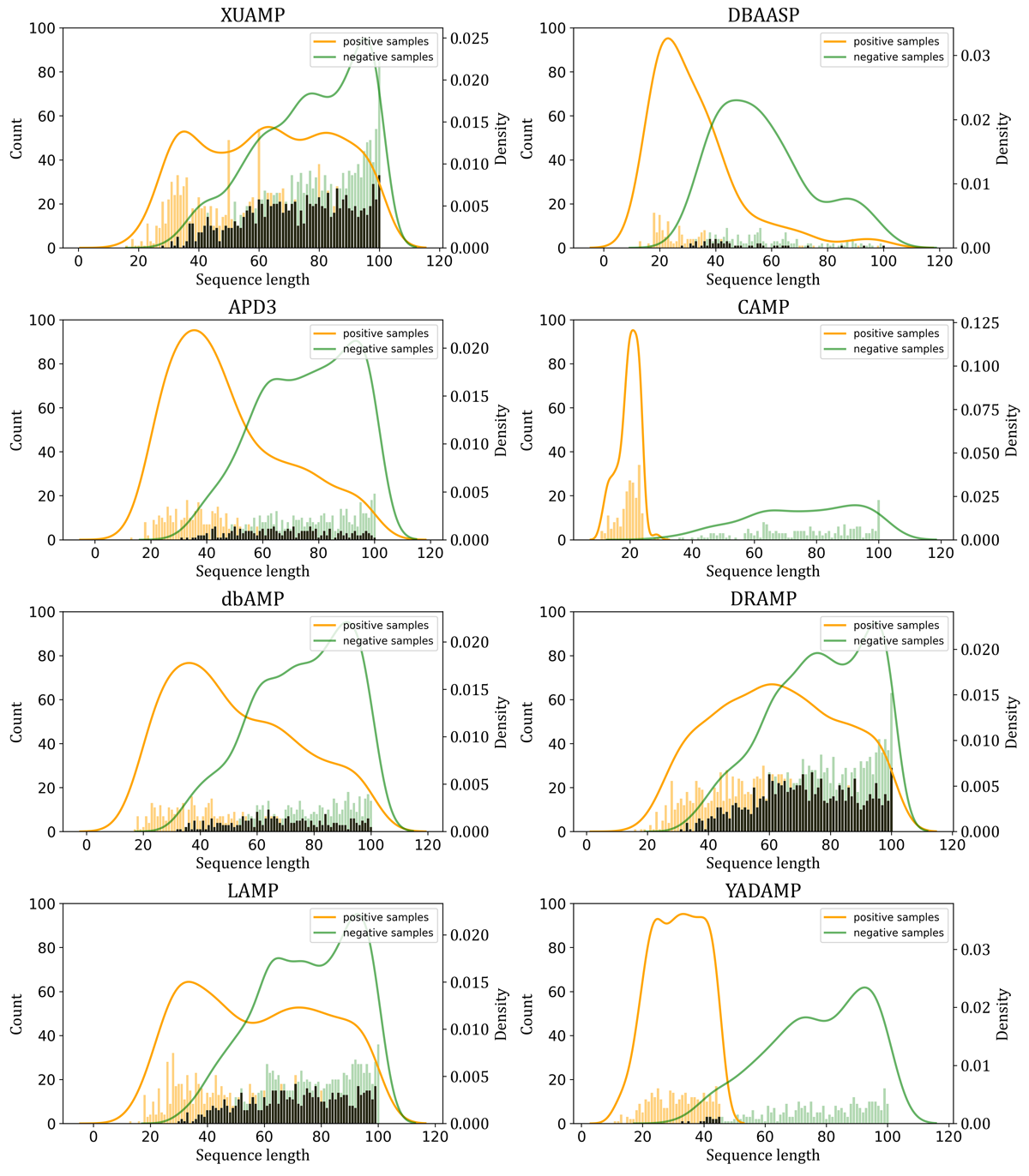

Supplement: btac715_Supplementary_Data [file btac715_supplementary_data.zip › btac715_Supplementary_Data/Fig s1.tif]

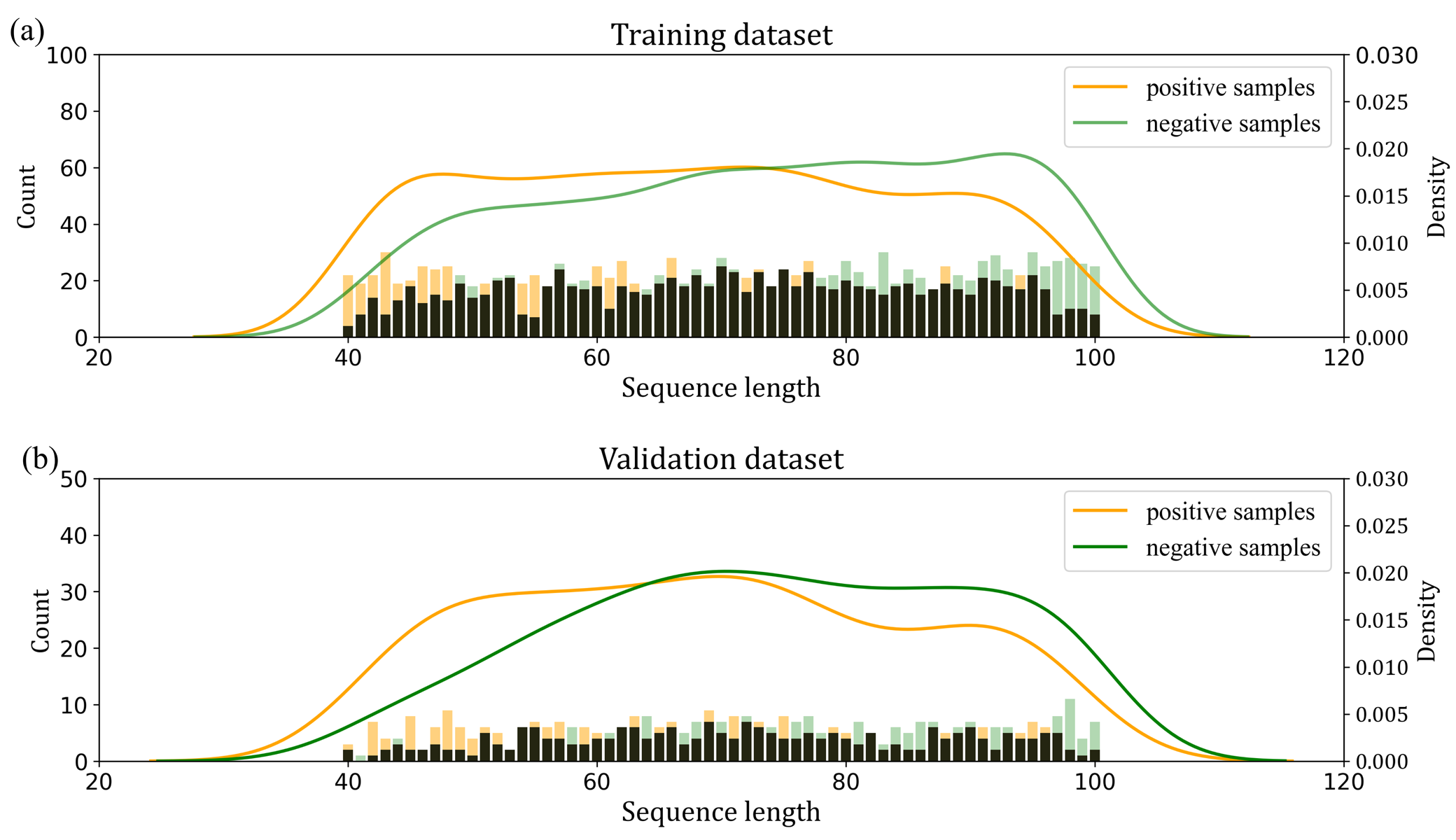

Supplement: btac715_Supplementary_Data [file btac715_supplementary_data.zip › btac715_Supplementary_Data/Fig s2.tif]

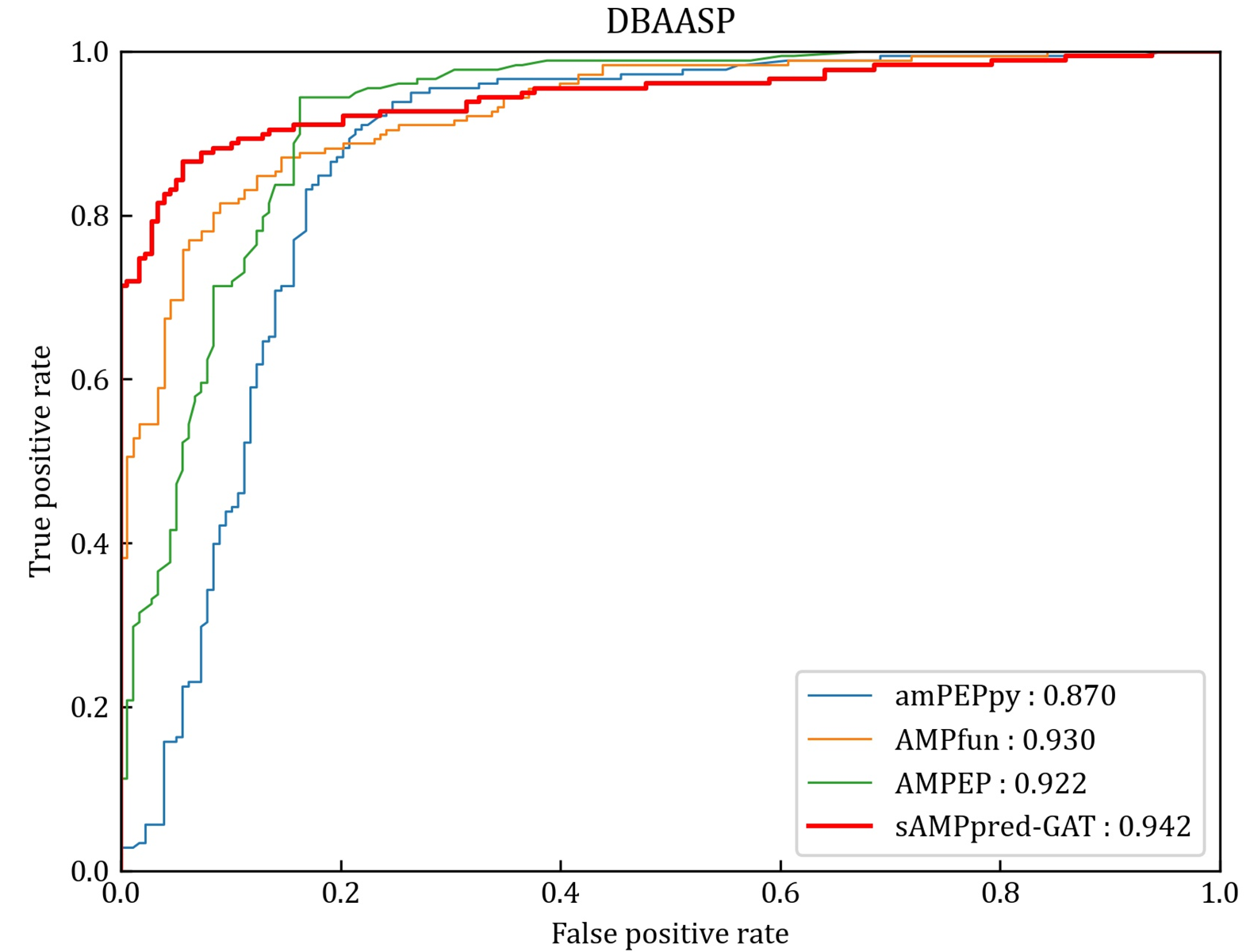

Supplement: btac715_Supplementary_Data [file btac715_supplementary_data.zip › btac715_Supplementary_Data/Fig s3.tif]
